# The Apollo Number: Space Suits, Self-Support, and the Walk-Run Transition

**DOI:** 10.1371/journal.pone.0006614

**Published:** 2009-08-12

**Authors:** Christopher E. Carr, Jeremy McGee

**Affiliations:** Massachusetts Institute of Technology, Cambridge, Massachusetts, United States of America; Universidad Europea de Madrid, Spain

## Abstract

**Background:**

How space suits affect the preferred walk-run transition is an open question with relevance to human biomechanics and planetary extravehicular activity. Walking and running energetics differ; in reduced gravity (<0.5 g), running, unlike on Earth, uses less energy per distance than walking.

**Methodology/Principal Findings:**

The walk-run transition (denoted *) correlates with the Froude Number (Fr = v^2^/gL, velocity v, gravitational acceleration g, leg length L). Human unsuited Fr* is relatively constant (∼0.5) with gravity but increases substantially with decreasing gravity below ∼0.4 g, rising to 0.9 in 1/6 g; space suits appear to lower Fr*. Because of pressure forces, space suits partially (1 g) or completely (lunar-g) support their own weight. We define the Apollo Number (Ap = Fr/M) as an expected invariant of locomotion under manipulations of M, the ratio of human-supported to total transported mass. We hypothesize that for lunar suited conditions Ap* but not Fr* will be near 0.9, because the Apollo Number captures the effect of space suit self-support. We used the Apollo Lunar Surface Journal and other sources to identify 38 gait events during lunar exploration for which we could determine gait type (walk/lope/run) and calculate Ap. We estimated the binary transition between walk/lope (0) and run (1), yielding Fr* (0.36±0.11, mean±95% CI) and Ap* (0.68±0.20).

**Conclusions/Significance:**

The Apollo Number explains 60% of the difference between suited and unsuited Fr*, appears to capture in large part the effects of space suits on the walk-run transition, and provides several testable predictions for space suit locomotion and, of increasing relevance here on Earth, exoskeleton locomotion. The knowledge of how space suits affect gait transitions can be used to optimize space suits for use on the Moon and Mars.

## Introduction

How space suits affect the walk-run transition is an open question with relevance to human biomechanics and extravehicular activity (EVA), a critical component of future human planetary exploration. Locomotion in space suits carries significant metabolic cost, which limits the intensity and duration, and hence value, of EVA. Walking and running incur different metabolic costs, and in reduced gravity environments (<0.5 g) running, unlike on Earth, uses less energy per unit distance than walking [1]. This finding also applies during space-suited locomotion [2], [3]. Space suits adversely impact the metabolic cost of walking more severely than running, likely due to the spring-like nature of space suit pressure forces [3], [4]. Space suits also appear to affect the walk-run transition; thus, space suits influence the energy cost of movement by influencing gait as well as by how they modify the metabolic cost of walking or running. Furthermore, by understanding how space suits impact the walk-run transition we can gain insight into the nature of gait transitions.

Here we develop a theory about how space suits may affect the walk-run transition based on a new non-dimensional parameter denoted the Apollo number, test the theory using data from the Apollo lunar surface missions of 1969–74, and explore the implications of our findings for EVA performance and discuss several testable predictions. First we summarize a simple model relating to the walk-run transition and show that our theory is a simple generalization of the concept of partial body-weight suspension (PBWS), a standard technique for simulating reduced gravity that is also used in rehabilitation. Then, we identify examples of walking and running gaits during lunar exploration and use them to test whether the Apollo number captures the effects of gravity and space suit self-support.

### The Unsuited Walk-Run Transition

Humans appear to choose walking or running to minimize oxygen consumption at their current velocity [5]. The Froude number, a nondimensional quantity equal to the ratio of inertial to gravitational force, can be used empirically to predict the walk-run transition in bipeds and quadrupeds [5], [6] according to the principle of dynamic similarity [6], [7]. For a body in an environment with gravitational acceleration 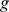, velocity 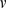, and hip height or center of mass height 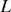, the Froude number can be written as
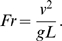
(1)


Modeling walking as an inverted pendulum [8] yields the constraint that walking can only occur for 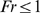 (Text S1).

The walk-run transition, which we denote by *, has generally been observed to occur in humans near 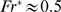 [6], consistent with the maximum walking speed constraint of the idealized inverted pendulum model. While the model does not predict a particular value for 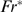, dynamic similarity [7] predicts constant 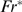 despite changes in 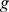 and 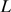, all other factors being equal, and this prediction is independent of any particular model of walking [9].

To understand how gravity effects 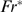 and to gain insight into locomotion energetics, studies have used partial body-weight suspension (PBWS) to simulate reduced gravity by applying a relatively constant upward force on the center of mass using a harness [1], [10], [11], [12]. These studies report Froude numbers based on the effective gravity level 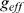, so that 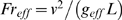. Here, 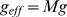, where 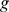 is the actual gravitational acceleration and 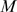 is the ratio of human supported to total transported mass (supplemental materials).

Kram et al. [11] found unsuited 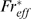 changed little from simulated reduced gravity levels down to 0.4 g, consistent with dynamic similarity, but increased to 1.1 as 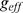 decreased further to 0.1 g; Kram et al. [11] did not measure 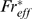 at lunar gravity (∼1/6 g), but interpolation yields 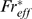 = 0.9. Kram et al. [11] attributed part of the increase in 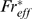 below 0.4 g to imperfection in the simulation method, but measurements of 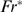 in true reduced gravity conditions during parabolic flight on board NASA's C-9 aircraft suggest that PBWS may be more accurate than previously assumed [13].

Substantially reduced gravity, then, appears to elevate 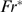, although 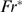 matches predictions of dynamic similarity over a greater than two-fold change in 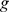. Space suits, in contrast, appear to decrease 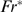, although controlled experiments involving running in space suits are rare, in part because 1 g space-suited running requires metabolic rates above the lactate threshold [2].

### Space Suit Self-Support

Just as PBWS affects the ratio of human carried to total transported mass 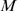, so does a space suit: internal pressure forces may support part or all of the space suit weight (supplemental materials). Rewriting 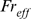 in terms of a Froude number involving the true gravitational acceleration and the mass ratio 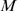 gives us a new quantity that we define as the “Apollo number” or 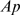 (Text S1):
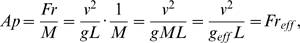
(2)with the same idealized restriction for walking of 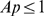.

In the case of no space suit, 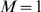 and 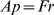. If the only space suit-related factor affecting the walk-run transition is 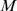, the fraction of total mass carried by the human, then the Apollo Number at the walk-run transition should be equal in value whether suited or unsuited. In this situation, the walk-run transition depends directly on the ratio of inertial to net gravitational force. This hypothesis is identical to the proposition that 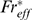 is constant across simulated gravity levels (supplemental materials), which appears to be a good approximation for 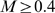 [11].

Therefore, for space-suited lunar locomotion, we hypothesize that 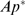 will be closer to the unsuited 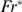 (∼0.9) than will 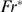, because the Apollo Number captures the effect of space suit self-support. Stated in an alternative manner, dynamic similarity predicts that space suits will reduce 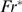 by about 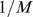 relative to unsuited 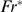.

## Methods

The Froude and Apollo numbers depend upon physical characteristics including leg length and body mass, so we assembled these data for the astronauts who explored the lunar surface (Table 1).

**Table 1:** Apollo Lunar Surface Astronaut Characteristics.

ID	Mission	Role*	Last Name	Mass†	Height‡	L§
				kg	m	m
1	11	CDR	Armstrong	76.2	1.80	0.97
2	11	LMP	Aldrin	75.5	1.78	0.96
3	12	CDR	Conrad	66.8	1.69	0.91
4	12	LMP	Bean	66.3	1.77	0.95
5	14	CDR	Shepard	76.4	1.80	0.97
6	14	LMP	Mitchell	80.1	1.80	0.97
7	15	CDR	Scott	79.6	1.83	0.99
8	15	LMP	Irwin	72.0	1.73	0.93
9	16	CDR	Young	77.2	1.75	0.95
10	16	LMP	Duke	71.8	1.82	0.98
11	17	CDR	Cernan	78.2	1.83	0.99
12	17	LMP	Schmidt	73.9	1.75	0.95

* Roles: Commander (CDR) and Lunar Module Pilot (LMP).

† Body mass estimated as mean of F-0 (Flight Day) and R+0 (Return Day) masses in Table 16 of Biomedical Results of Apollo [25].

‡ Height from astronaut biographies in the Apollo 11–17 Press Kits, available from the Apollo Lunar Surface Journal [14].

§ Leg length L estimated as Height/1.85, following [2].

We exhaustively reviewed audio transcripts and video clips of lunar EVAs available from the Apollo Lunar Surface Journal [14], and several NASA technical reports related to lunar surface locomotion [15], [16], [17] to identify gait events on the lunar surface for which we had some evidence of the gait type (walk/lope/run) and could estimate locomotion velocity (see below). Using the locomotion velocities and subject characteristics we estimated 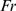 for each event.

**Figure 1:**
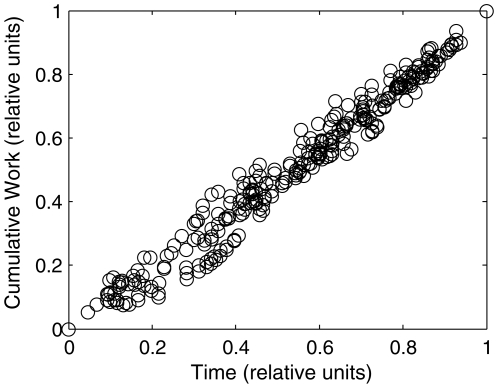
Cumulative metabolic expenditures during Apollo lunar surface exploration can be approximated as linear with time. Using the original Apollo metabolic rate data tables [4], cumulative metabolic expenditures (joules) were estimated for each astronaut for each EVA and mission, and were normalized, with unity representing the end-of-EVA condition. Linear fits within each EVA-mission-astronaut condition (not shown) had minimum adjusted R^2^>0.97; 20 of 27 conditions (74%) had R^2^>0.99.

**Figure 2:**
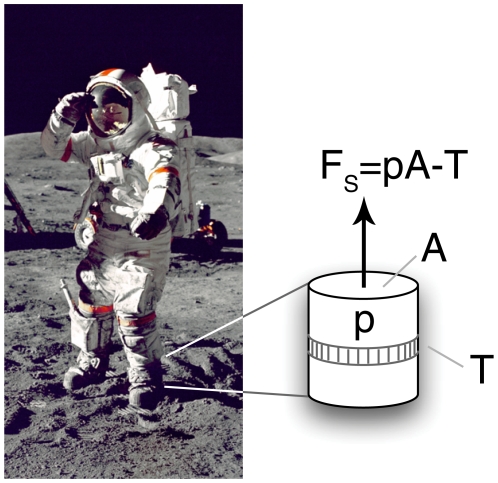
Model of space suit self-support. Self-support force 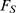, in an idealized model of the Apollo space suit in a vacuum, is set by the product of suit pressure 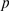 and minimum cross sectional area 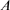 minus the tension 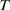 in the restraint layer (supplemental materials). Image: Eugene Cernan during Apollo 17. NASA/Harrison Schmitt.

We took total transported mass as the sum of body mass and estimated suit mass at the time of each event. We approximated the space suit mass based on the suit type (A7L, used for Apollo 11–14, or the A7LB, the latter used for the Apollo 15–17 missions) and by assuming a constant consumables usage rate during each EVA (Table 2), an accurate approximation based on subsequent analysis of metabolic rates (Figure 1). Space suit self-support, modeled as an idealized pressurized column, is limited by the minimum cross section, found at the ankle joint (Figure 2). We assumed complete space suit self-support based on calculations using space suit ankle joint measurements (Nicole Jordan, personal communication) and video data of lunar astronauts (Text S1, Video S1). After estimating 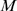, we computed 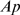 for each gait event.

**Table 2:** Characteristics of Lunar Surface EVAs.

ID*	Start MET†	Stop MET†	Duration	Suit‡	Use Rate§
	hhh∶mm∶ss	hhh∶mm∶ss	hours		kg/hour
11.1	108∶56∶02	111∶41∶28	2.76	A7L	4.83
12.1	115∶08∶02	119∶09∶05	4.02	A7L	3.31
12.2	131∶29∶27	135∶22∶57	3.89	A7L	3.42
14.1	113∶36∶49	118∶26∶01	4.82	A7L	2.76
14.2	131∶06∶32	135∶42∶05	4.59	A7L	2.90
15.1	119∶37∶06	126∶10∶34	6.56	A7LB	4.01
15.2	142∶13∶19	149∶26∶14	7.22	A7LB	3.64
15.3	163∶18∶19	168∶05∶55	4.79	A7LB	5.48
16.1	118∶52∶00	126∶03∶50	7.20	A7LB	3.65
16.2	142∶38∶06	150∶01∶55	7.40	A7LB	3.55
16.3	165∶30∶28	171∶10∶37	5.67	A7LB	4.64
17.1	117∶00∶53	124∶13∶40	7.21	A7LB	3.64
17.2	140∶32∶49	149∶11∶09	8.64	A7LB	3.04
17.3	163∶31∶45	170∶47∶12	7.26	A7LB	3.62

*ID A.B is Apollo Mission A, and EVA #B during Mission A.

† Mission Elapsed Times (METs) for EVA start and stop based on Lunar Module depressurization/repressurization times.

‡ Dry/wet masses: 66.8/81.6 kg (A7L) or 66.8/96.0 kg (A7LB).

§ Estimated mean consumables use rate assuming full at start of EVA and 10% safety margin at end of EVA.

On Earth, humans commonly use only walking and running gaits, but on the Moon, astronauts used a variety of hopping-like gaits often referred to in prior studies as loping. Loping, often like skipping but without the support-foot exchange [18], shares features of walking and running [18], [19].

To analyze astronaut gait in terms of a binary transition, we assigned a binary variable *gait* (walk or lope = 0, run = 1) for each event. We analyzed the variable *gait* using a standard logit model (methods) to estimate the transition probability 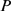 (probability that gait = 1) for both 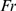 and 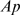 and determine the estimated walk-run transition, or point where 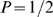.

Finally, to facilitate comparisons at lunar gravity, we fit unsuited 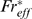 values from Kram et al. [11] using the power law 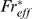 = C(G)^k^, and found C = 0.42±0.04, k = −0.42±0.06 (mean±95%CI), with an adjusted R^2^ = 0.98.

### Gait Type and Velocity Estimation During Lunar EVA

Allowable evidence for the gait type included specific mention of gait type in the audio transcripts or associated written commentary in [14], observation of gait type via video clip, or written description of the gait type in the case of the NASA technical reports [15], [16], [17], which were written specifically to analyze human movement on the lunar surface.

**Figure 3:**
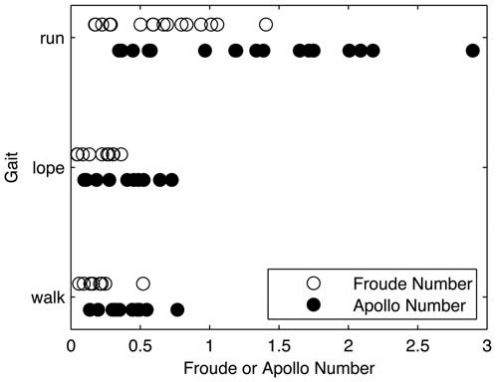
Gait events during Apollo lunar surface EVA. Walking and loping have similar 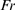 and 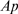 distributions; running conditions were associated with higher velocities than either walking or loping conditions. For details of each condition see supplemental materials (Table S1).

Velocities derived from the Apollo Lunar Surface Journal [14] and the NASA technical reports [15], [16], [17] are based on extensive reconstructions of astronaut time and distance measurements, the latter frequently determined from calibrated images of the lunar surface. We did not extract time and position data from videos but relied upon prior measurements of known time and position. For example, gait condition 10 (Table S1) is based on video determination of gait type and velocity determination from commentary in [14] that reads: “Neil's run across the TV picture takes about 25 seconds. According to Figure 3–16 in the Apollo 11 Preliminary Science Report, the distance he covered in this time is 22 meters. His running speed is, therefore, about 3.2 km/h [0.89 m/s].”

All of the videos analyzed and the source for video S1 are in the public domain and are not copyrighted (image credits: NASA/Ken Glover).

### Space Suits are Self-Supporting in Lunar Gravity

Imagine an inflatable column, torque stabilized so that it cannot buckle prematurely; the maximum mass supported by this stabilized column, an idealized approximation of a space suit leg, is given by 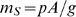, where 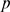 is the differential pressure across the column wall, 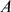 is the minimum cross section, and 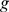 is the gravitational acceleration acting on the mass.

For a space suit in a vacuum (Figure 2), 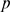 is the internal suit pressure, and 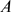 is the cross-sectional area of the ankle joint (the point of minimum cross sectional area), so that the force supported by a single space suit leg is 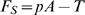, where 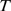 is the tension in the load-bearing “skin” of the space suit, known as the restraint layer, at the cross section. For high suit weights (e.g. on Earth) or low pressures , the maximum net force 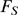 occurs when 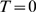 so that 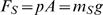, and the self-support is partial (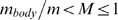, where 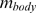 is the mass of the astronaut, and 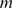 is the total transported mass). Because the maximum net force 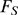 cannot exceed the suit weight, at low suit weights (e.g. on the Moon) or high pressures, the maximum net force is equal to the suit weight and self-support is complete (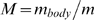).

Consider the pressure forces transmitted by a single space suit leg with a minimum cross section diameter of 14.6 cm or 5.75 inches. This value represents the approximate ankle ring inner diameter of the current NASA EMU space suit and the approximate diameter of the most narrow ankle cross section of the Apollo A7LB suit, which had no ankle ring (unpublished observations, Nicole Jordan). For a suit pressure of 26.2 kPa (3.8 psi) and minimum cross sectional area of 168 cm^2^ (26.0 in^2^), the pressure force of 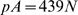 exceeds the 156N lunar weight of the A7LB at its maximum mass condition (96 kg) by a factor of 2.8. Thus, a space suit on the lunar surface can be considered entirely self-supporting at the walk-run transition, where the time-averaged number of legs in contact with the ground is approximately one.

Direct evidence of this assertion is provided by videos of the Apollo astronauts demonstrating the challenge of reaching the lunar surface: the high pressure forces (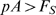 so that 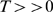) made it challenging to fully buckle the knee joint, even when standing on one leg. In one case (Apollo 16, 146∶49∶41, Video S1) an astronaut can be seen jumping into the air in an attempt to provide (during the following impact) enough force (through body weight and impact loads) to buckle the knee joint and reach a hammer on the lunar surface.

### Logit Transition Model

Generalized linear models (GLMs) relate the random distribution of a measured variable to a linear predictor though a link function, the appropriate choice of which depends upon the distribution of the measured variable. In our case, the measured variable is *gait* (walk/lope = 0, run = 1), and the predictor variable 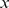 is 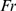 or 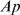. Because gait is binomial, the proper canonical link function is the logit. For probability 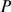, the odds ratio is given by 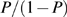, and the logit transformation defined as 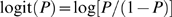. Here, 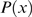 is the probability that *gait* = 1, and can be expressed as
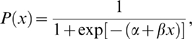
which has the convenient property that 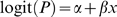, making this widely applicable model also easy to fit. The two parameters 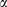 and 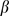 describe the shape and location of the state transition, with the transition point 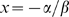 defined by 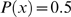.

The data were fit using the MATLAB GLM fitting function *glmfit()* (The Mathworks, Natick, MA), which calculates the parameters 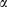 and 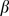, the variance estimates 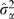 and 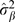, and the covariance 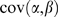. The standard error of the transition point was estimated as
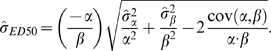



## Results and Discussion

We identified and analyzed 38 classifiable gait events (Figure 3, Table S1) with mean 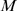 = 0.49. Of these events, 10 involved walking, 10 loping, and 18 running. Walking and loping generally occurred at lower Froude or Apollo numbers than running; there was no significant difference between the mean 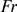 for walking and loping (two-tailed t-test, p = 0.95). We pooled walking and loping data on the basis of two considerations: First, loping Froude numbers are statistically indeterminate from those of walking. Second, walking and loping share the exchange of kinetic and potential energy of the center of mass that is absent in running.

**Figure 4:**
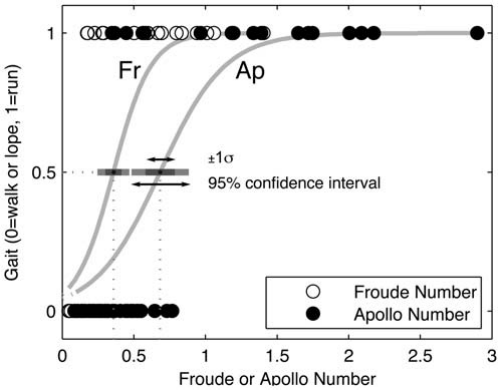
Gait transition probability as a function of Froude and Apollo numbers. Transition probability curves 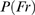 and 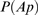 for the binary variable *gait* (walk/lope = 0, run = 1). Here, 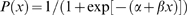, with the transition defined by 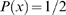, where 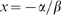 (Text S1). For values of 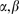 see Table 3.

**Figure 5:**
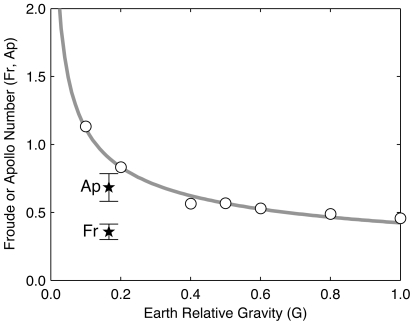
Reduced gravity and the preferred walk-run transition. Simulated reduced gravity has little effect above but a large effect below 0.4 g on the walk-run transition. Unsuited walk-run transitions Froude Numbers (open circles) are well fit by a power law (gray line). Transitions (labeled 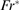 and 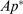) determined in this study are denoted by stars (mean±s.d.). Unsuited 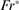 data from Kram et al. [11]. See text for details.

All logit parameters for fits to 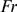 and 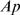 data (Figure 4) were significant (Table 3), and the walk-run transitions for 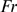 (0.36±0.11, mean±95%CI) and 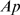 (0.68±0.20) were significantly different (p≪0.001). Our estimated lunar suited 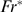 and 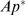 values were 60% and 24% lower, respectively, than the estimated lunar unsuited 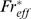 = 0.90 from the Kram et al. [11] power law fit (Figure 5, gray line; G = g/g_earth_).

**Table 3:** Logit Fit Results.

Fit	Logit Parameter Values*			p-values†	
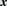	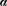	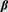	transition	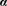	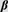
Fr	−2.75±0.79	7.68±2.40	0.358±0.057	0.0014	0.0028
Ap	−3.04±0.88	4.45±1.47	0.684±0.102	0.0015	0.0046

*Logit parameter values are mean ±s.d.

† All significant at 95% level (p<0.05).

As a rough test of our hypothesis, we conclude that the Apollo number is closer to the unsuited Froude number (∼0.9) than the (suited) Froude number. Similarly, the 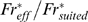 ratio of 2.51 differs from the expected value of 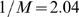 by 23%.

Kram et al. [11] adjusted 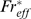 to account for the downward inertial force caused by the swing leg (which experienced 1 g forces that would not be present in true reduced gravitational environments), causing their adjusted 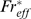 values to range from 0.39 in 1 g to 0.67 in 0.1 g. However, experiments in NASA's C-9 aircraft, which produces the closest Earth-analog to lunar gravity by flying modified parabolic flight profiles, have measured unsuited 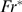 = 1.39±0.45 (mean±s.d., N = 8) [13]. Thus, the aforementioned adjustment may represent a substantial over-correction. G-level fluctuations during parabolic flight, and the short period (∼30 s) of lunar gravity available per trial may contribute to the high measured 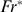; for example, slightly lower g-levels could produce higher 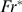 estimates because the 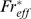 vs. gravity curve is quite steep near 1/6 g (Figure 5). However, an experiment on NASA's POGO [13], a high-fidelity (2–10% dynamic error) pneumatic controlled partial body-weight suspension device [20], found unsuited 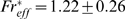 (mean±s.d., N = 4).

Taking the unadjusted Kram et al. [11] data as representative of true unsuited 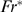, the Apollo Number explains 60% of the difference between the lunar suited 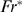 (0.36) and lunar unsuited 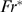. If NASA POGO estimates (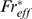
∼1.22) are more representative, then the Apollo Number explains 38% of this difference. Thus, changes in the walk-run transition speed in suited versus unsuited locomotion appear attributable, at least in part, to space suit self-support.

**Figure 6:**
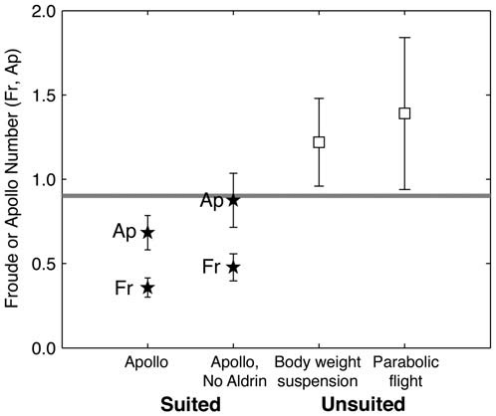
Walk-run transition parameters in lunar gravity. Apollo (lunar, suited) 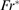 and 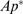 points (stars) are from this study (full and restricted datasets, respectively); unsuited transition 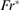 (open squares) are from Hagan et al. [13] as described in the text. All values are mean±s.d. The gray horizontal line represents the expected unsuited 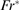 in lunar gravity, interpolated from data in Kram et al. [11].

Inherent limits to the dataset restrict the fidelity of our analysis: For example, conditions 1–9 (Table S1) are derived from a single three-minute period during astronaut Aldrin's gait and mobility evaluation (a prime objective of Apollo 11), and may admit the highest risk of subject bias of any set of gait events in Table 2. Fitting a restricted dataset, without conditions 1–9, results in 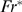 = 0.478±0.158 (mean±95%CI) and 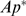 = 0.876±0.314, suggesting that the Apollo Number may explain more of the observed difference in the suited and unsuited 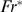 (e.g. up to 94% based on Kram et al. [11] data) than our initial analysis indicated (Figure 6).

Unsurprisingly, the Apollo Number does not completely explain the observed difference: space suits may impact the walk-run transition speed through factors other than self-support, and our assumption of perfect self-support is itself an approximation. It is unknown whether gait transitions are triggered via metabolic signals [5], by muscle force production or activation [21], or by other factors such as stability [22]. Space suits may also influence the walk-run transition through mobility restriction, increased joint mechanical work due to joint torques (generated in large part from pressure-volume work resulting from non-constant volume joints), changes in mass distribution and thus stability, and other as yet unquantified factors such as leg stiffness changes that may modify gait dynamics. Finally, although all gait conditions had 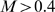, the gravity dependence of 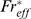 suggests that for 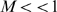 the Apollo number may not fully capture the effect of space suit self support (supplemental materials).

Despite these limitations, our theory of invariant 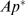 despite manipulations of loading (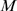) provides several testable predictions. First, at higher suit masses with continued full self-support (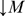), the walk-run transition will occur at a lower speed (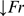). Because running in a space suit has a lower cost of transport (energy/distance) than walking [2], [3], lowering the walk-run transition may provide energetic benefits that permit expansion of the region able to be explored during an EVA. In an idealized model, the “walkback” restriction allows exploration of the region defined by a circle with a radius, determined by remaining consumables (oxygen, CO2 scrubbing capability, cooling water), that shrinks with time. Space-suited running may have low cost of transport, but absolute metabolic rates are still high (for example, 326W and 429W for two running conditions during Apollo 16 [16]). Under conditions of full self-support, large suit mass may reduce the walk-run transition speed, facilitating efficient locomotion at lower and more sustainable metabolic rates.

Second, when carriage of large loads reduces self-support (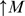), walking becomes possible at higher velocities (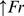). The only walking condition involving a heavy load (Table S1, condition 36) occurred during transport of the 116 kg Apollo Lunar Surface Experiments Package via carry-bar to its deployment site. For this (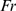 = 0.52, 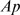 = 0.77) condition, 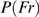 = 0.78 and 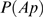 = 0.59 (restricted dataset: 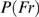 = 0.61, 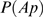 = 0.34). This is consistent with a near constant 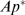, where the condition represents a gait near (technically slightly above) the run walk transition; this condition is completely concordant with the higher 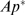 of the restricted dataset, where it would be expected to represent a walk. However, the condition is above 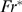 and the condition's 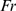 = 0.52 is significantly elevated (one-sided z-test, z = 5.52, p≪0.001) relative to all other walking conditions, none of which included similar loads. The data support the theoretical increase in 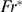 with increased human-supported load fraction (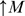).

A third prediction relates to changes in the walk-run transition when humans use exoskeletons with external load paths, such as those under consideration for load-carrying [23] or those used for medical rehabilitation [24]. Consider a human wearing an exoskeleton that supports an additional body weight equivalent of mass, so that 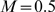. For an unsuited 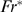
∼0.5, we might expect (taking L = 0.95 m) a walk-run transition velocity near v*∼2.2 m/s (4.8 mph). With the exoskeleton (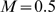) we might now expect 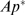
∼0.5, 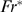
∼0.25, and v*∼1.5 m/s (3.4 mph), a rather slow and potentially energetically inefficient running velocity in 1 g. Experimental verification that external load paths modify 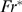 in the expected manner has been demonstrated using a lower-body exoskeleton designed to simulate the knee joint-torques of the current NASA spacesuit (C. Carr, unpublished observations). Knowing the walk-run transition and its energetic consequences during exoskeleton locomotion could be useful in determining the range of transport speeds consistent with efficient exoskeleton usage, and may guide the design of exoskeletons, such as inclusion of high energy-return springs optimized for a particular speed, frequency, or range of motion.

In summary, we have developed a theory of how 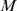, the ratio of human supported to total transported mass, affects the walk-run transition and tested this theory using gait events from space-suited lunar locomotion. The Apollo Number (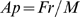) appears to explain a significant part of the difference between the unsuited and suited walk-run transition Froude numbers, and as expected, space-suited 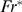 is well below the unsuited 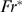. Several predictions can now be tested: if our theory is correct, manipulation of self-support, whether by changes in space suit pressure or mass, gravitational environment, or via exoskeleton load carrying, will change the walk-run transition speed but have little effect on the Apollo Number. Indeed, PBWS experiments have shown that humans have near constant 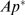 for moderate reductions in simulated gravity, and that exoskeletons with external load paths lower the walk-run transition speed.

## Supporting Information

Text S1 (0.22 MB DOC)

Table S1 Classifiable Gait Events During Lunar Locomotion
(0.16 MB DOC)

Video S1 Apollo space suits self-support in lunar gravity. During this scene from Apollo 16, Astronaut Charles Duke drops a hammer on the lunar surface, then jumps repeatedly in order to overcome the self-support of the space suit by compressing the space suit knee joint(s) so that he might retrieve the hammer from the surface.
(4.85 MB MOV)
