# Supplementary material for: The Apollo Number: Space Suits, Self-Support, and the Walk-Run Transition
Source: PLoS One. 2009 Aug 12;4(8):e6614. doi: 10.1371/journal.pone.0006614 (PMC2719915; doi:10.1371/journal.pone.0006614)
Supplement: Text S1 — (0.22 MB DOC) [file pone.0006614.s001.doc]

# The Apollo Number: Space suits, self-support, and the walk-run transition

Christopher E. Carr[[1]](#footnote-2)*, Jeremy McGee

Massachusetts Institute of Technology, Cambridge, MA, USA

**Text S1**

# Nomenclature

$v$ Average forward locomotion velocity (speed) [m/s]

$g$ Gravitational acceleration [m/s^2^]

$L$ Leg length or approximate center of mass height [m]

Froude number, [nondimensional]

* Denotes walk-run transition, e.g. denotes Froude number at the walk-run transition

$m$ Total mass transported, sum of body and space suit mass [kg]

$m_{\text{body}}$ Body mass [kg]

$m_{s}$ Mass supported by space suit pressure forces, partial body weight suspension device, or external load path of exoskeleton [kg]

$M$ Ratio of human-carried to total transported mass or load, $M=(m-m_{S})/m$ [nondimensional]

$g_{\text{eff}}$ Effective gravitational acceleration during partial body weight suspension, $g_{\text{eff}}=\text{Mg}$ [m/s^2^]

$\text{Fr}_{\text{eff}}$ Froude number based on effective gravitational acceleration, [nondimensional]

Apollo number, $\text{Ap}=\text{Fr}/M$ [nondimensional]

Binary variable indicating gait form (walk/lope=0, run=1)

$P(x)$ Probability that $\text{gait}=1$ for predictor variable $x$ = or

$\alpha$,$\beta$ Parameters that specify the shape and transition point of the best-fit logit model for $P(x)$

$R^{2}$ Standard Pearson correlation coefficient

$p$ Space suit differential pressure, internal minus external [N/m^2^]

$A$ Minimum cross sectional area of the space suit (e.g. ankle joint) [m^2^]

$T$ Tension in the restraint layer of the space suit at the minimum cross section [N]

$F_{S}$ Self-support force due to space suit pressure forces, $F_{S}=\text{pA}-T$, or support force provided during partial body weight suspension, [N]

$\hat{s}_{\alpha}^{2}$,$\hat{s}_{\beta}^{2}$ Variance estimates for logit transition model parameters $\alpha$ and $\beta$

# Model Derivation

**Standard Walk-Run Transition Model**

For a body in an environment with gravitational acceleration $g$, velocity $v$, mass $m$, and characteristic length $L$, the Froude number can be written as:

(S1)

When applied to human locomotion, the characteristic length $L$ is usually taken as the height of the hip joint, which is approximately equal to the height of the center of mass. The Froude number is proportional to the ratio of kinetic to potential energy of the center of mass: Kinetic energy of a moving body (neglecting internal kinetic energy) is $\frac{1}{2}\text{mv}^{2}$, whereas potential energy relative to the surface is .

In ballistic walking models [1], which approximate walking as the motion of an inverted pendulum, gravity provides the force required to counter-balance the inertial force ${\text{mv}^{2}}/L$, and the condition for the stance leg to remain on the ground is:

, (S2)

which becomes:

, (S3)

or in terms of the Froude number,

. (S4)

**Space Suit Self-Support**

A space-suited astronaut does not support the entire mass of the space suit during locomotion, because part or all of the mass is supported by the pressure forces of the space suit (see Methods section entitled *Space Suits are Self-Supporting in Lunar Gravity*, Video S1). We denote the mass supported by the space suit as the *self-support* mass, $m_{S}$.

The reduction in total load carried by the human reduces the available counter force in ballistic walking: the centripetal force remains ${\text{mv}^{2}}/L$, but now the available counter force is only , where $F_{s}$ is the net pressure force resulting from space suit pressurization. Following equation (2), the static condition for the stance leg to remain on the ground is given by

. (S5)

This can be rewritten as

, (S6)

which is now recognizable as the product of the Froude number and a mass ratio equal to the reciprocal of the fraction of mass carried directly by the human. Defining

, (S7)

and defining the “Apollo Number” as:

(S8)

we can now write the condition for the stance leg to remain on the ground as:

. (S9)

**Partial Body-Weight Suspension**

Here we show that partial body-weight suspension (PBWS) is directly analogous to our idealized theory of space suit self-support, and that the hypothesis of constant Apollo number across loading conditions is equivalent to constant Froude numbers across simulated reduced gravity conditions.

Standard PBWS studies use a harness or other apparatus to approximate reduced gravity through application of an approximately constant vertical suspension force F_S_ so that the effective weight is:

, (S10)

giving an effective gravitational acceleration of

, (S11)

where $m$ is the total transported mass and $g$ is the actual gravitational acceleration. Froude numbers in PBWS studies are usually reported in terms of effective gravity level, e.g. . Noting that the effective gravitational acceleration is also defined by the ratio of human carried to total transported mass $M$, so that $g_{\text{eff}}=\text{Mg}$, we can write:

, (S12)

Thus, our hypothesis that Ap is constant across loading conditions is equivalent to the hypothesis that is constant across simulated reduced gravity conditions, a necessary condition for dynamic similarity [2]. Kram et al. [3] found that is nearly constant from effective g-levels from 0.4-1g, but increases at simulated g-levels below 0.4. Thus, we might expect that is approximately constant for . Finally, our hypothesis is reasonable because all of the gait conditions we evaluated (Table S1) have .

# References

1. Mochon S, McMahon TA (1980) Ballistic walking: an improved model. Mathematical Biosciences 52: 241-260.

2. Bullimore SR, Donelan JM (2007) Criteria for dynamic similarity in bouncing gaits. Journal of Theoretical Biology doi:10.1016/j.jtbi.2007.09.038.

3. Kram R, Domingo A, Ferris DP (1997) Effect of reduced gravity on the preferred walk-run transition speed. Journal of Experimental Biology 200: 821-826.

4. Jones E (2006) Apollo Lunar Surface Journal, NASA History Website.

5. Kubis J, Elrod J, Rusnak R, Barnes J (1972) Apollo 15 time and motion study. National Aeronautics and Space Administration.

6. Kubis J, Elrod J, Rusnak R, Barnes J, Saxon S (1972) Apollo 16 time and motion study. National Aeronautics and Space Administration.

7. Kubis J, Elrod J, Rusnak R, McLaughlin E, Moseley E (1970) Analysis of Apollo XI lunar EVA (Mobility Evaluation). In: Manned Spacecraft Center H, Texas, editor: National Aeronautics and Space Administration.

1. ^*^ Corresponding author. Current position and affiliation: Research Scientist, MIT Department of Earth, Atmospheric and Planetary Sciences. Mailing address: Massachusetts Institute of Technology, 77 Massachusetts Ave, Room 54-418. Cambridge, MA 02139 USA. Tel: +1-617-253-0861. Email address: chrisc@mit.edu. [↑](#footnote-ref-2)
