# Supplementary material for: The Apollo Number: Space Suits, Self-Support, and the Walk-Run Transition
Source: PLoS One. 2009 Aug 12;4(8):e6614. doi: 10.1371/journal.pone.0006614 (PMC2719915; doi:10.1371/journal.pone.0006614)
Supplement: Table S1 — Classifiable Gait Events During Lunar Locomotion (0.16 MB DOC) [file pone.0006614.s002.doc]

Table S1. Classifiable Gait Events During Lunar Locomotion

| ID | Mission | EVA | Subject | MET | Velocity | Fr | M* | Ap | Gait | | Source† |
| --- | --- | --- | --- | --- | --- | --- | --- | --- | --- | --- | --- |
|  |  |  |  | hhh:mm:ss | m/s |  |  |  |  |  |  |
| 1 | 11 | 1 | 2 | 110:13:15 | 0.52 | 0.17 | 0.50 | 0.35 | Run | 1 | B |
| 2 | 11 | 1 | 2 | 110:13:37 | 0.67 | 0.29 | 0.50 | 0.58 | Run | 1 | B |
| 3 | 11 | 1 | 2 | 110:13:47 | 0.66 | 0.28 | 0.50 | 0.56 | Run | 1 | B |
| 4 | 11 | 1 | 2 | 110:14:21 | 0.59 | 0.22 | 0.50 | 0.45 | Run | 1 | B |
| 5 | 11 | 1 | 2 | 110:14:27 | 0.52 | 0.17 | 0.50 | 0.35 | Run | 1 | B |
| 6 | 11 | 1 | 2 | 110:14:46 | 0.64 | 0.26 | 0.50 | 0.53 | Lope | 0 | B |
| 7 | 11 | 1 | 2 | 110:15:03 | 1.02 | 0.67 | 0.50 | 1.34 | Run | 1 | B |
| 8 | 11 | 1 | 2 | 110:15:29 | 0.96 | 0.59 | 0.50 | 1.18 | Run | 1 | B |
| 9 | 11 | 1 | 2 | 110:15:46 | 1.04 | 0.69 | 0.50 | 1.39 | Run | 1 | B |
| 10 | 11 | 1 | 1 | 111:10:49 | 0.89 | 0.50 | 0.52 | 0.97 | Run | 1 | A |
| 11 | 12 | 1 | 4 | 118:22:46 | 1.11 | 0.80 | 0.48 | 1.65 | Run | 1 | A |
| 12 | 12 | 1 | 3 | 118:22:46 | 1.11 | 0.83 | 0.49 | 1.72 | Run | 1 | A |
| 13 | 12 | 1 | 4 | 118:24:32 | 1.25 | 1.01 | 0.48 | 2.09 | Run | 1 | A |
| 14 | 12 | 1 | 3 | 118:24:32 | 1.25 | 1.06 | 0.49 | 2.18 | Run | 1 | A |
| 15 | 12 | 2 | 4 | 132:12:43 | 1.11 | 0.80 | 0.46 | 1.75 | Run | 1 | A |
| 16 | 14 | 1 | 5 | 154:41:10 | 0.97 | 0.60 | 0.50 | 1.19 | Run | 1 | A |
| 17 | 15 | 1 | 7 | 120:44:00 | 0.37 | 0.09 | 0.46 | 0.18 | Lope | 0 | C |
| 18 | 15 | 1 | 8 | 120:49:00 | 0.30 | 0.06 | 0.44 | 0.14 | Walk | 0 | C |
| 19 | 15 | 1 | 8 | 120:50:00 | 0.27 | 0.05 | 0.44 | 0.11 | Lope | 0 | C |
| 20 | 15 | 1 | 7 | 121:59:00 | 0.46 | 0.13 | 0.48 | 0.28 | Lope | 0 | C |
| 21 | 15 | 1 | 8 | 123:30:00 | 0.46 | 0.14 | 0.47 | 0.30 | Walk | 0 | C |
| 22 | 15 | 2 | 8 | 144:28:00 | 0.58 | 0.22 | 0.45 | 0.50 | Walk | 0 | C |
| 23 | 15 | 2 | 8 | 144:28:00 | 0.61 | 0.25 | 0.45 | 0.55 | Walk | 0 | C |
| 24 | 15 | 2 | 7 | 144:33:00 | 0.27 | 0.05 | 0.47 | 0.10 | Lope | 0 | C |
| 25 | 15 | 2 | 7 | 144:33:00 | 0.58 | 0.21 | 0.47 | 0.44 | Walk | 0 | C |
| 26 | 15 | 3 | 8 | 165:31:11 | 0.37 | 0.09 | 0.46 | 0.20 | Walk | 0 | C |
| 27 | 15 | 3 | 8 | 165:33:11 | 0.58 | 0.22 | 0.46 | 0.48 | Walk | 0 | C |
| 28 | 16 | 1 | 10 | 124:11:03 | 0.53 | 0.18 | 0.48 | 0.37 | Run | 1 | D |
| 29 | 16 | 2 | 9 | 144:59:48 | 0.68 | 0.30 | 0.47 | 0.64 | Lope | 0 | D |
| 30 | 16 | 2 | 10 | 146:35:04 | 0.60 | 0.23 | 0.47 | 0.49 | Lope | 0 | D |
| 31 | 16 | 2 | 10 | 149:19:17 | 0.76 | 0.36 | 0.50 | 0.73 | Lope | 0 | D |
| 32 | 16 | 3 | 10 | 167:15:59 | 0.48 | 0.14 | 0.45 | 0.32 | Walk | 0 | D |
| 33 | 16 | 3 | 10 | 167:16:19 | 0.50 | 0.16 | 0.45 | 0.35 | Walk | 0 | D |
| 34 | 16 | 1 | 10 | 120:40:21 | 0.70 | 0.31 | 0.68 | 0.45 | Lope | 0 | D |
| 35 | 16 | 1 | 10 | 120:40:52 | 0.66 | 0.27 | 0.68 | 0.40 | Lope | 0 | D |
| 36 | 16 | 1 | 10 | 120:44:47 | 0.91 | 0.52 | 0.68 | 0.77 | Walk | 0 | D |
| 37 | 17 | 1 | 12 | 120:17:51 | 1.20 | 0.94 | 0.47 | 2.01 | Run | 1 | A |
| 38 | 17 | 1 | 11 | 120:36:04 | 1.50 | 1.40 | 0.49 | 2.90 | Run | 1 | A |

*Assumes full suit self-support ( estimated suit mass at indicated time) and linear model of consumables usage, e.g. constant consumable use rate during EVA.

†Sources: A: Apollo Lunar Surface Journal (14), B: Analysis of Apollo XI lunar EVA (Mobility Evaluation) (17), C: Apollo 15 time and motion study (15), D: Apollo 16 time and motion study (16).
